# Multiplicity of *Plasmodium falciparum *infection following intermittent preventive treatment in infants

**DOI:** 10.1186/1475-2875-9-244

**Published:** 2010-08-26

**Authors:** Ulrike Buchholz, Robin Kobbe, Ina Danquah, Philipp Zanger, Klaus Reither, Harry H Abruquah, Martin P Grobusch, Peter Ziniel, Jürgen May, Frank P Mockenhaupt

**Affiliations:** 1 Institute of Tropical Medicine and International Health, Charité - University Medicine, Berlin, Germany; 2 Infectious Disease Epidemiology Group, Bernhard Nocht Institute for Tropical Medicine, Hamburg, Germany; 3 Institute of Tropical Medicine, University of Tuebingen, Tuebingen, Germany; 4 Swiss Tropical and Public Health Institute, Basel, Switzerland; 5 Division of Microbiology and Infectious Diseases, University Hospital, Kwame Nkrumah University of Science and Technology, Kumasi, Ghana; 6 Medical Research Unit, Hôpital Albert Schweitzer, Lambaréné, Gabon; 7 Department of Infectious Diseases, Tropical Medicine, HIV/AIDS, Amsterdam Medical Center, University of Amsterdam, The Netherlands; 8 Northern Region Malaria Project, Tamale, Ghana; 9 Dept. of Immunology & Microbiology, Rush University, Chicago, USA

## Abstract

**Background:**

Intermittent preventive treatment in infants with sulphadoxine-pyrimethamine (IPTi-SP) reduces malaria morbidity by 20% to 33%. Potentially, however, this intervention may compromise the acquisition of immunity, including the tolerance towards multiple infections with *Plasmodium falciparum*.

**Methods:**

*Plasmodium falciparum *isolates were obtained from children participating in two Ghanaian IPTi-SP trials (Tamale, Afigya Sekyere) at 15 months of age, i.e., six months after they had received the second dose of IPTi-SP or placebo. By typing the polymorphic *merozoite surface protein 1 *(*msp1*) and *msp2 *genes, multiplicity of infection (MOI) was assessed in 389 isolates. A total of additional 133 samples were collected in Tamale at 3, 6, 9, and 12 months of age. Comparisons of MOI between groups were done by non-parametric statistical tests.

**Results:**

The number of distinguishable *P. falciparum *clones (MOI) ranged between one and six. Mean MOI in Tamale was stable at 2.13 - 2.17 during the first year of life, and increased to 2.57 at age 15 months (*P *= 0.01). At no age did MOI differ between the IPTi-SP and placebo groups (each, *P *≥ 0.5). At 15 months of age, i.e., six months after the second dose, MOI was very similar for children who had received IPTi or placebo (means, 2.25 *vs*. 2.33; *P *= 0.55) as was the proportion of polyclonal infections (69.6% vs. 69.7%; *P *= 0.99). Adjusting for study site, current and prior malaria, parasite density, and season did not change this finding.

**Conclusions:**

IPTi-SP appears to have no impact on the multiplicity of infection during infancy and thereafter. This suggests that tolerance of multiple infections, a component of protective immunity in highly endemic areas, is not affected by this intervention.

## Background

Efficient and safe means of controlling falciparum malaria in endemic areas are urgently needed to reduce its intolerable high burden, particularly in young children. Reducing parasite exposure, however, may compromise the acquisition of protective immunity, as has been debated with respect to vector control and was observed following chemoprophylaxis [1-3].

Multiplicity of infection (MOI) denotes the number of distinguishable *Plasmodium falciparum *clones *per *isolate. Commonly, MOI is assessed by using the polymorphic regions of *P. falciparum *merozoite surface proteins 1 and 2 genes (*msp 1/2*) as markers. In that, inter-laboratory differences exist [4], and methods displaying higher parasite diversity have been developed [5]. Irrespective of that, MOI has been considered to be subject to and to form part of the complex immune mechanisms in malaria. In children intensely exposed to antigenically diverse parasites, MOI is considered to roughly indicate the extent of acquired immunity by reflecting tolerance to multiclonal infections. These, in turn, may stimulate and maintain cross-protective immune responses [6,7]. The association between MOI and the subsequent risk of malarial disease is intricate and depends on, e.g., age and transmission intensity. At intense transmission, MOI increases in parallel to the acquisition of immunity and to the ability to control parasitaemia [6]. Indeed, among older children living in these areas, high MOI has repeatedly been associated with protection from subsequent clinical episodes [7-12]. In contrast, *P. falciparum *infections of comparatively short duration in infants preclude tolerance [13], and multiclonal infections rather increase the risk of clinical attacks [14-16]. The latter is also observed in areas of moderate transmission [7,16,17].

Intermittent preventive treatment in infants (IPTi) denotes the administration of a curative dose of an anti-malarial, commonly sulphadoxine-pyrimethamine (SP) at the times of routine vaccination, regardless of whether a child is parasitaemic or not. IPTi with SP (IPTi-SP) was initially tested in Tanzania also as an approach to avoid the rebound effect of increasing morbidity following chemoprophylaxis. In that initial study, IPTi-SP reduced the incidence of malaria in infancy by 59% without evidence of a rebound effect [18]. In five subsequent trials using slightly different treatment schedules, this protective efficacy ranged between 20% and 33% [19-23]. Overall, IPTi-SP in these six trials in Tanzania, Mozambique, Ghana and Gabon had a protective efficacy against clinical malaria of 30% in the first year of life [24]. Evidence for a rebound of malaria (high density parasitaemia, severe malaria) in the second year of life was observed in two studies [19,22]. Meanwhile, IPTi has been tested at further sites and using different drugs [25,26], and WHO considers IPTi for implementation in areas of moderate to high transmission and where SP resistance is not high [27].

So far, only scarce data are available on the effect of anti-malarial interventions on MOI. In Tanzania, MOI was significantly reduced among infants infected despite chemoprophylaxis and this reduction was suggested to be involved in the subsequent rebound in clinical malaria episodes [28]. RTS, S vaccination reduced MOI in Mozambique and Kenya [29,30] whereas insecticide-treated nets (ITNs) left MOI virtually unchanged in Tanzania [31]. Here, in two Ghanaian IPTi cohorts, a potential sustained impact of IPTi on multiclonal infections was examined by assessing MOI in infected children six months after they received their second dose of IPTi-SP.

## Methods

*Plasmodium falciparum *isolates obtained from children participating in two related, randomized IPTi trials conducted in Tamale, hyperendemic northern Ghana, and in Afigya Sekyere district, holoendemic southern Ghana, were examined. The attempt of identical examinations in the IPTi trial from Lambaréné, Gabon [23], was abandoned because of scarce availability of positive samples precluding a meaningful analysis. The study protocols were reviewed and approved by the Ethics Committees, University for Development Studies, Tamale, Kwame Nkrumah University of Science and Technology, Kumasi, and International Foundation of the Albert Schweitzer Hospital, Lambaréné, and informed consent was obtained from the children's parents. Details of the site characteristics and the analysed two trials have been presented elsewhere. The trial in Tamale was performed from March 2003 through July 2005, and the one in Afigya Sekyere from January 2003 through September 2005 [21,22]. Briefly, while Tamale is a city of some 350,000 inhabitants but of rather rural character, the study in Afigya Sekyere comprised nine villages of 1,500 to 13,000 inhabitants. Malaria transmission is perennial with seasonal variation at both sites; entomologic data are not available for Tamale but in Afigya Sekyere, approximately 400 infective bites per person-year were observed in 2003-2005 (unpublished data). Coverage with insecticide-treated bed nets was low at ≤3% in both sites in 2003-2005 [21,22]. In the first year of life, the incidence of malaria has been estimated as 0.95 and 1.27 episodes per person-years at risk in Tamale and Afigya Sekyere, respectively [24]. In the two IPTi trials, 2270 children were recruited at three months of age and followed up actively in three-monthly (Tamale) or monthly (Afigya Sekyere) intervals, and passively, until 24 months of age. IPTi-SP or placebo was administered at ages 3, 9, and 15 months. Active follow-up included clinical examination, regular blood sampling, and parasitological and haematological examinations. During passive follow-up visits, i.e. when patients presented independently from regular visits, identical procedures were done as detailed elsewhere [21,22]. Episodes of malaria were treated with artesunate (4 mg/kg, double dose on first day, for five days, Tamale) or amodiaquine-artesunate (10/4 mg/kg for three days, Afigya Sekyere). A sample size of ≥1,070 infants in each trial was estimated to provide 80% power for detecting a 20% reduction of hazards of developing malaria in the SP group, compared with the placebo group [21,22].

On the day of IPTi dose-3, i.e., six months following IPTi dose-2 and at approximately 15 months of age, venous blood samples were collected. These were stabilized by adding an equal volume of DNA-stabilizing buffer (AS1, Qiagen, Germany), stored at +4°C, and DNA was extracted by commercial kits (QIAmp, Qiagen, Germany). All samples with microscopically confirmed *P. falciparum *parasitaemia were subjected to assessing MOI. For that, sequences corresponding to the allelic families of *P. falciparum msp1 *block 2 (K1, Mad20, Ro33) and of *msp2 *block 3 (FC27, IC) were amplified in five separate nested PCR assays [32]. These alleles are characterized by conserved regions flanked by repeat sequences of variable length. Therefore, size variation within the alleles can be used to discriminate different parasite clones by PCR fragment length polymorphism. Fragments were separated on 3% GTG^®^-agarose gels (Biozym, Germany) and analysed using GeneSnap software (SynGene, UK). In case of negative or inconclusive PCR results, assays were repeated maximally twice. MOI was calculated as the highest number of fragments for either *msp1 *or *msp2*. Of a total of 348 microscopically positive samples collected at 15 months of age, MOI could be assessed in 277 (79.6%). For the Tamale trial, all samples collected at 15 months of age were examined by *Plasmodium*-specific nested PCR assays [33] and m*sp1/2 *genotyping was extended to additional 142 samples identified to have submicroscopic parasitaemia of which 112 yielded a result for MOI (typing efficiency, 78.9%). Also, for the Tamale cohort, a random sample of microscopically positive samples (if available, each 25 for placebo and IPTi-SP groups) collected at 3, 6, 9, and 12 months of age was genotyped to illustrate the development of MOI with age (typing efficiency, 72.2%). For all samples, processing and DNA extraction were performed in a standardized manner, and all samples were typed at the Institute of Tropical Medicine in Berlin, Germany.

Malaria was defined by the presence of microscopically visible parasitaemia *plus *fever (axillary temperature, ≥37.5°C, or, Afigya Sekyere, ≥38.0, <12 months, rectally; ≥12 months, tympanically) or fever during the preceding 48 hours reported by mothers without being asked. For incidence estimates, children were regarded as not being at risk for 21 days following an episode of malaria and person-time-at-risk was reduced accordingly. Polyclonal infections had a MOI of >1. Rainy season (> 10 days or >90 mm of precipitation/month) was defined as before [5,22].

Continuous parameters were compared between groups by the non-parametric Mann-Whitney U-test or Kruskal-Wallis test. These tests were also applied to compare MOI between groups but results are primarily presented as arithmetic means since this measure of average more selectively displays (the direction of) differences than medians, which are also shown, nevertheless. Proportions of polyclonal infections were analysed by χ^2 ^tests. In multivariate analysis, known or potential confounders of the proportion of polyclonal infections and MOI were adjusted for by including these in logistic regression models and by applying non-parametric multiple ordinal regression, respectively.

## Results

The characteristics of the 1086 children at 15 months of age from Tamale and their 964 peers from Afigya Sekyere are shown in Table 1. As compared to children from Tamale, their peers from Afigya Sekyere showed more microscopically determined parasitaemia, more frequently had a current episode of malaria but less frequently were febrile or anaemic. Also, more children from Afigya Sekyere than from Tamale had had malaria until 15 months of age, and the number of previous episodes was higher. Most children in Tamale had their scheduled visit at 15 months of age during the rainy season, but only roughly half in Afigya Sekyere (Table 1).

**Table 1:** Characteristics of 2050 Ghanaian children at 15 months of age, i.e. six month after the third dose of IPTi-SP

Parameter	Tamale	Afigya Sekyere	*P*
No.	1086	964	
Proportion girls (%)	50.2	49.3	0.68
Examination for present study (15 months of age) in rainy season (%)	89.6	49.3	< 0.0001
SP group : placebo group	542 : 544	485 : 479	0.86
Current *P. falciparum *infection			
by microscopy (%; n/n)	11.0 (119/1084)	24.3 (229/944)	< 0.0001
GMPD (parasites/μL, 95%CI)	2312 (1599-3344)	1455 (1105-1917)	0.03
by PCR (%)	23.6 (248/1049)	n.a.	-
Current malaria episode (%, n/n)	7.4 (80/1084)	11.3 (107/944)	0.002
Febrile (%, n/n)	12.8 (138/1082)	6.1 (59/964)	< 0.0001
History of fever within 48 h (%, n/n)	31.7 (344/1086)	31.8 (307/964)	0.93
Haemoglobin (g/dL; mean, range)	8.91 (4.7-12.7)	9.90 (2.0-15.0)	< 0.0001
Proportion anaemic (Hb < 11 g/dL; %, n/n)	94.0 (1019/1084)	74.4 (715/961)	< 0.0001
Proportion severely anaemic (Hb < 7 g/dL; %, n/n)	8.0 (87/1084)	4.9 (47/961)	0.004
Proportion with prior episode(s) of malaria (%, n/n)	47.4 (515/1086)	57.6 (555/964)	< 0.0001
No. of previous malaria episodes (mean, range)	0.67 (0-5)	1.23 (0-7)	< 0.0001

GMPD, geometric mean parasite density; Hb, haemoglobin; n.a., not available

In Tamale and Afigya Sekyere, IPTi-SP at ages 3 and 9 months had achieved reductions in the incidence of malaria until 15 months of age of 24% (13-33%) and 20% (95%CI, 12-28%), respectively, largely due to incidence reductions during the first month following each treatment [21,22,24]. At 15 months of age, no impact of previous IPTi-SP on malariometric parameters was discernible: children who had received IPTi-SP or placebo showed similar prevalences of asymptomatic parasitaemia (16.9% (172/1015) *vs*. 17.4% (176/1013), *P *= 0.80) and malaria (9.6% (97/1015) *vs*. 8.9% (90/1013), *P *= 0.60). Also, GMPDs (95%CI) were alike (1622 (1175-2238) *vs*. 1791 (1318-2432)/μL, *P *= 0.66).

**Figure 1:**
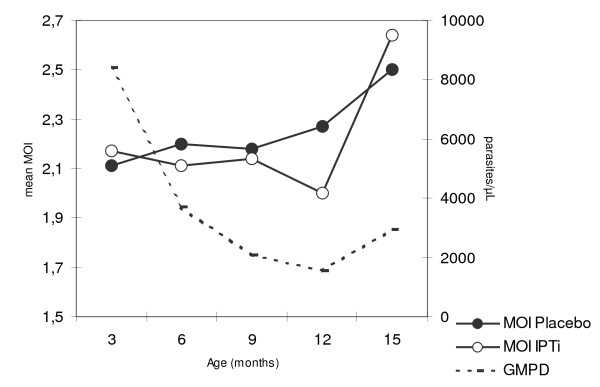
**Multiplicity of infection and geometric mean parasite density according to age**. Group sizes at ages 3, 6, 9, 12, 15 months: 15, 38, 44, 36, 104. Parasite density declines until 12 months of age and slightly increases thereafter (*P *= 0.08). IPTi had no effect on parasite density (*P *= 0.57), at no time (*P *> 0.4). In multiple, non-parametric ordinal regression analysis, IPTi had no effect on MOI in these children (regression coefficient, -0.33; standard error (SE), 0.33; *P *= 0.32; age (months), 0.06, SE, 0.06; *P *= 0.30; log10 parasite density, 0.78, SE, 0.19; *P *< 0.0001).

The development of MOI with age in Tamale is shown in Figure 1. Overall, MOI was stable at a mean of 2.13 - 2.17 (medians, each 2) during the first year of life (*P *= 1.0), and increased to 2.57 (median, 2; range 1-5) at age 15 months (*P *= 0.01). At no age, did MOI differ between IPTi-SP and placebo groups (*P *≥ 0.5). Lacking impact of IPTi-SP on MOI was confirmed when adjusting for parasite density and age by multiple ordinal regression (*P *= 0.32; Figure 1).

**Figure 2:**
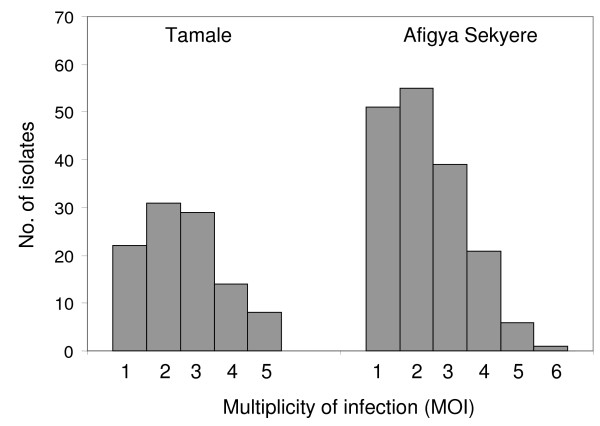
**Distribution of MOI in Tamale and Afigya Sekyere**.

At 15 months of age, a total of 277 samples with microscopically visible parasitaemia were successfully genotyped, comprising 163 (58.8%) samples of malaria cases. The distribution of MOI in these 277 samples is shown in Figure 2. Further 112 specimens of submicroscopic infections were available from Tamale. Irrespective of study site, MOI (mean, 2.29; median, 2; range, 1-6) and the proportion of polyclonal *P. falciparum *infections (69.7%) were similar among former IPTi-SP and placebo recipients. This was true for both microscopically identified and submicroscopic infections (Table 2).

**Table 2:** Multiplicity of infection and proportion of polyclonal infections according to previous IPTi with SP

Stratum	**No**. IPTI : placebo	mean MOI (median, range)		*P*	Proportion of polyclonal infections (%)		*P*
					
		Previous IPTi	Previous Placebo		Previous IPTi	Previous Placebo	
All	191 : 198	2.25 (2, 1-5)	2.33 (2, 1-6)	0.55	69.6	69.7	0.99
All microscopically positive	135 : 142	2.42 (2, 1-5)	2.38 (2, 1-6)	0.78	74.8	72.5	0.67
Afigya Sekyere	85 : 88	2.29 (2, 1-5)	2.31 (2, 1-6)	0.92	69.4	71.6	0.75
Tamale, all	106 : 110	2.22 (2, 1-5)	2.35 (2, 1-5)	0.49	69.8	68.2	0.80
Tamale, microscopically positive	50 : 54	2.64 (2.5, 1-5)	2.50 (2, 1-5)	0.59	84.0	74.1	0.22
Tamale, submicroscopic	56 : 56	1.84 (2, 1-5)	2.20 (2, 1-5)	0.17	57.1	62.5	0.56

*P*, comparisons by Mann-Whitney U test or χ^2 ^test

Known factors influencing multiplicity and potential confounders were tested (Table 3): MOI (and polyclonal infections) in microscopically positive samples were increased in children with current malaria, tended to be higher in Tamale than in Afigya Sekyere, and increased with parasite density (Table 3). Prior occurrence of malaria was not associated with MOI itself but, at borderline significance, with less polyclonal infections. A lowered multiplicity of *P. falciparum *in the rainy season was observed only in multivariate analysis. Adjusting for these factors, the finding that previous IPTi-SP did not influence the multiplicity of *P. falciparum *infection was confirmed (Table 3).

**Table 3:** Univariate and multivariate associations with microscopically visible, polyclonal *P. falciparum *infections

Parameter	**No**.	Mean MOI (median, range)	*P*	**Multivariate analysis**^**a**^			Polyclonal infections (%)	OR (95%CI)	*P*	**Multivariate analysis**^**b**^	
								
				b	SE	*P*				aOR (95%CI)	*P*
Previous IPTi-SP											
No	142	2.38 (2, 1-6)					72.5	1		1	
Yes	135	2.42 (2, 1-5)	0.78	-0.05	0.22	0.82	74.8	1.12 (0.64-1.99)	0.67	0.93 (0.52-1.64)	0.79
Cohort											
Tamale	104	2.57 (2, 1-5)					78.8	1		1	
Afigya Sekyere	173	2.30 (2, 1-6)	0.07	-0.67	0.26	0.01	70.5	0.64 (0.35-1.18)	0.13	0.53 (0.28-1.03)	0.06
Current malaria episode											
No	114	2.21 (2, 1-6)					64.0	1		1	
Yes	163	2.53 (2, 1-5)	0.01	0.55	0.24	0.02	80.4	2.30 (1.29-4.11)	0.002	2.35 (1.27-4.32)	0.006
Parasite density (/μL)											
Log10	277	n.a.	0.049	0.24	0.13	0.07	n.a.	1.48 (1.08-2.03)	0.01	1.38 (0.99-1.94)	0.06
Prior malaria episodes											
None	72	2.50 (2, 1-5)					81.9	1		1	
Any	205	2.37 (2, 1-6)	0.30	-0.19	0.26	0.46	70.7	0.53 (0.26-1.09)	0.06	0.51 (0.25-1.05)^c, d^	0.07
Season											
Dry season	92	2.49 (2, 1-5)					79.3	1		1	
Rainy season	185	2.36 (2, 1-6)	0.32	-0.77	0.27	0.004	70.8	1.58 (0.84-3.01)	0.13	0.36 (0.18-0.72)	0.004

^a^, multivariate results are derived from non-parametric ordinal regression analysis including all listed parameters. ^b^, adjusted odds ratios (aORs) are derived from logistic regression models including all other listed parameters; SE, standard error; OR, odds ratio; 95%CI, 95% confidence interval; n.a., not applicable; ^c^, exchanging with number of previous malaria episodes (0-7): aOR, 0.98, 95%CI, 0.81-1.19; *P *= 0.84. ^d^, The proportion of polyclonal infections according to arbitrarily set time periods from last previous episode was: <8 weeks (*n *= 65), 76.9%; 8-16 weeks (*n *= 62), 72.6%; >16 weeks (*n *= 78), 64.1%. Exchanging this graded parameter with prior episodes *per se *yielded respective aORs (95%CIs) of 0.73 (0.30-1.78), 0.72 (0.29-1.76), and 0.32 (0.14-0.73).

In the Tamale cohort, submicroscopic infections showed a lesser multiplicity than those microscopically visible (mean MOI, 2.02 *vs*. 2.57; medians (range), each 2 (1-5); *P *= 0.0006). Submicroscopic infections were arbitrarily set as 1 parasite/μL and included into a separate multivariate analysis as above for the Tamale cohort. Again, no significant impact of IPTI-SP on MOI (regression coefficient, -0.15; standard error, 0.36; *P *= 0.67) or on polyclonal infections (adjusted OR, 1.03; 95%CI, 0.56-1.87, *P *= 0.93) was discernible.

Between months 16 and 24 of follow-up of the Tamale cohort, 12 excess cases of severe malaria were observed in the previous IPTi group [22]. For the six of these 12 children for whom genotyping data were available at 15 months of age, MOI did not differ from the remaining 100 children in the IPTi group (means, 2.00 *vs*. 2.23; medians (range), 2 (1-5) *vs*. 2 (1-3); *P *= 0.82).

## Discussion

In holoendemic Tanzania, continuous chemoprophylaxis in infancy reduced the multiplicity of infection with *P. falciparum*, and the rebound of malaria following that intervention has been attributed to this reduction [28]. In the present analysis of two IPTi-SP cohorts from hyper- and holoendemic Ghana, no evidence was found that this intervention has a sustainable effect on multiplicity. This finding supports the principle idea of IPTi, i.e. a basically chemoprophylactic intervention of limited duration [21,22,24,34] allowing for sufficient exposure to develop and maintain immunity. In consequence also, because IPTi-SP did not affect multiplicity which has been linked to prospective risks of malaria [7-17] this study does not support the attribution of potential rebound effects to interference with MOI. In particular, in the Tamale cohort, rebound events in the former IPTi-SP recipients were not associated with MOI at 15 months. Admittedly, however, only few samples were available for analysis, and most children with later severe malaria were not infected at that time.

The present study analysed multiplicity only, i.e. one actor in the network of diverse immune mechanisms. The importance of this factor is furthermore varying with age and endemicity [7-17]. Also, the single assessment of MOI at 15 month of age may provide only a still image of an actually dynamic parasite composition [35]. Data were derived exclusively from infected children, i.e., the minority of individuals under study. Typing efficiency was at the lower range of previously reported results [4]. Thus, a proportion of children who were infected, presumably at very low parasite densities, could not be included into the analysis. The risk of malaria at a given time is determined by a multitude of factors including among others, host heterogeneity and temporal variation in exposure, genetics, socio-economic status, and treatment-seeking behaviour [36,37] which, however, due to randomization should be evenly distributed between the placebo and IPTi groups. Thus, as one limitation of the present study, the results are valid only for those children harbouring parasites on examination, and the influence of other factors on malaria and malarial immunity may outweigh the one of multiplicity of infection.

The present data accord with previous findings from highly endemic areas [13,14] in that MOI was basically stable throughout the first year of life and increased only at its end. Paralleled by the increasing ability to control parasitaemia (Figure 1) this may indicate a change of the predominant type of host response at this age. While in infancy infections are rather short-lived [13] and fever and pro-inflammatory responses are the main defence armamentarium [38], the development of tolerance to the establishment and persistence of (low-grade) parasitaemia, i.e. premunition, is considered a prerequisite for clinical immunity [6]. However, it should be noted MOI is also interpreted alternatively, namely as a parameter of (recent) accumulation of infections, higher exposure, and possibly increased malaria incidence rather than a proxy parameter for premunition [16,35]. Obviously, MOI is a highly complex parameter and subject to various inter-related influences. Of note, the temporal dynamics of *P. falciparum *infection also affect the assessment of parasite populations, and single assessments may provide only a limited impression of the overall parasite composition in a host [35]. In the present study, MOI increased with parasite density likely reflecting known threshold effects in the detection of individual parasite clones [14,16,35]. The observed increased multiplicity in clinical episodes of malaria accords with some previous reports [14,39,40] but contrasts with others [41,42]. Considering young age and endemicity, the finding of the present study that MOI is increased in malaria might result from the recent acquisition of novel parasite clones (as reflected by high MOI) which has been reported to increase the probability of simultaneous clinical episodes [13,39]. Unexpectedly, MOI was lower in the rainy than in the dry season. Despite possible distortion of results by delayed onset of increased transmission, this contrasts with previous studies [32,43,44] but is in line with a study among Ghanaian infants in whom the variability of circulating allelic populations was higher in the dry season [5]. Although heterogeneity of malaria transmission, i.e. pockets of high transmission during the dry season, might be involved in a comparatively high MOI among children infected in the dry season, the actual reasons for this are, however, obscure. Prior malaria episodes rather reduced the odds of current multiclonal infection. However, the proportion of polyclonal infections did not differ between children with no previous or recent malaria, and among those with prior episodes, there was a trend for less polyclonal infections the longer the last episode dated back (Table 3). Thus, considering that short-lived immune mechanisms evoked by an infection and controlling (rather than eliminating) multiple infections form the backbone of the concept of premunition [6], this finding may reflect a temporal decline in the ability to tolerate infection following an episode. Lastly, multiplicity tended to be higher in Tamale than in Afigya Sekyere although the opposite was expected [7,16,17,43]. Possibly, a *de facto *higher incidence of malaria has been missed in Tamale due to the comparatively broader mesh of active follow-up. Also, the use of artesunate for treatment there as compared to long-acting artesunate-amodiaquine in Afigya Sekyere might be involved in a more intense exposure.

## Conclusions

IPTI-SP did not influence the multiplicity of *P. falciparum *up to the age of 15 months, although it reduced the incidence of malaria in the two trials by 24% and 20%. This effect on malaria could largely be attributed to a prophylactic effect of approximately one month duration following treatment [21,22,24]. This resembles the situation with ITNs, i.e., morbidity reduction and absent effect on multiplicity [31] suggesting that imperfect or short-lived interventions allow for sufficient exposure. *Vice versa*, the reduction of MOI following RTS, S vaccination [29,30] might reflect its long-lasting activity. IPTi-SP is debated controversially [24,45,46], reasons including the absence of effect in areas of high SP resistance [25], potential contributions to the emergence of drug resistance [47], and clues for rebound effects [19,21,22]. As for the latter, no evidence was found suggesting that interference with multiplicity of infection could be involved as has been proposed for chemoprophylaxis [28]. The present study also highlights that MOI is influenced by various factors which need to be taken into account when interpreting the respective effects of interventions. The impact of IPTi on multiplicity of infection and the development of immunity using alternative, long-acting drugs which likely will replace SP in the future [25] needs to be defined.

## Competing interests

The authors declare that they have no competing interests.

## Authors' contributions

FPM, JM, and MPG designed the study. PZa, KR, SA, RK, HHA, and Pzi were responsible for patient recruitment, and clinical and parasitological examinations. UB did the genotyping, and ID and FPM the statistical analyses. UB, ID and FPM wrote the paper with major contributions of the other authors. All authors read and approved the final manuscript.
